# Effect of kombucha soymilk on high fat diet mice: integrated insights from gut microbiome and metabolome analyses

**DOI:** 10.3389/fnut.2026.1815305

**Published:** 2026-05-13

**Authors:** Yingyi Liu, Min Li, Mingkun Pan

**Affiliations:** 1 Department of Anesthesiology, China-Japan Union Hospital of Jilin University, Changchun, Jilin, China; 2 Department of Clinical Laboratory, China-Japan Union Hospital of Jilin University, Changchun, Jilin, China; 3 College of Pharmacy, Yanbian University, Yanji, Jilin, China

**Keywords:** gut microbiome, high fat diet mice, kombucha soymilk, linoleic acid, metabolome

## Abstract

**Introduction:**

Kombucha, soymilk, and tea-derived bioactive compounds have individually been associated with metabolic benefits, while the effects of kombucha soymilk on diet-induced hyperlipidemia and its associated gut microbiome–metabolome changes remain unclear.

**Methods:**

In this study, we established a high-fat diet (HFD)-induced obese mouse model and administered kombucha soymilk as a dietary intervention. We systematically investigated the effects on body weight gain, lipid levels, and hepatic antioxidant capacity, and further explored the associated changes in gut microbiota composition and key metabolites underlying its lipid-lowering effects.

**Results and discussion:**

Biochemical and histological analyses revealed that kombucha soymilk consumption significantly attenuated body weight gain in mice (p< 0.05), reduced serum and hepatic triglyceride (TG) and total cholesterol (TC) levels (p < 0.01), enhanced hepatic antioxidant capacity, and ameliorated hepatic steatosis. Microbiome analysis revealed that kombucha soymilk consumption altered the gut microbial community structure in mice, increasing the relative abundances of Enterococcus, Bifidobacterium, and Turicibacter. Untargeted metabolomics further suggested altered enrichment of pathways related to pyruvate metabolism, linoleic acid metabolism, bile secretion, and cAMP signaling. In conclusion, kombucha-fermented soymilk improved hyperlipidemia-related phenotypes in HFD-fed mice and was associated with selective gut microbial and metabolic alterations. These findings support its potential as a functional dietary intervention, although the mechanistic interpretation remains exploratory and requires further validation.

## 1 Introduction

Hyperlipidemia is a prevalent metabolic disorder characterized by elevated levels of cholesterol and/or triglycerides in the blood. As of 2020, the World Health Organization (WHO) reported that over 400 million people worldwide are affected by hyperlipidemia. The prevalence of this condition varies based on factors such as dietary patterns, lifestyle choices, and genetic factors (1).

Tea consumption has been linked to beneficial effects on hyperlipidemia. Clinical and murine studies consistently showed that tea intake significantly reduces circulating lipid levels, although the magnitude of these effects varies by tea type and individual differences (2, 3). Tea contains catechins and polyphenolic compounds, and both *in vitro* and *in vivo* murine studies have demonstrated that these compounds exert potent antioxidant activity, thereby lowering blood lipid levels, inhibiting cholesterol oxidation, and preventing plaque formation in vascular walls (4, 5). Additionally, both domestic and international studies using murine models have shown that tea intake may increase metabolic rate and promote fat oxidation, thereby reducing body fat accumulation (6, 7). Green tea is well known to be abundant in tea polyphenols and is widely recognized for its potential to ameliorate hyperlipidemia (8, 9). In murine models, Epigallocatechin gallate (EGCG) has been demonstrated to inhibit cholesterol absorption and promote its removal (10, 11). Various types of tea are also thought to exert beneficial effects in the management of hyperlipidemia, as their bioactive constituents can effectively reduce circulating levels of TC, TG, and total lipids (12). Additionally, tea can enhance liver function and metabolism in mice, contributing to the regulation of blood lipid levels (13, 14).

Soymilk exhibits significant regulatory effects on hyperlipidemia by lowering the levels of total TC, TG, and low-density lipoprotein cholesterol (LDL-C), while increasing high-density lipoprotein cholesterol (HDL-C) in humans (15). Human intervention studies indicates that consuming foods rich in plant sterols in a high-saturated-fat diet can achieve the effect of controlling blood pressure and reducing the long-term risk of cardiovascular diseases (16). Soybean milk fermented by LP-HFY01 attenuated the high-fat diet–induced increases in serum LDL-C, TG, alkaline phosphatase (ALP), and aspartate aminotransferase (AST) levels in mice, while preventing the decrease in HDL-C (17). Furthermore, soymilk yogurt fermented with *Pediococcus pentosaceus* TOKAI 759 m exhibits potential beneficial effects and ameliorates cognitive decline in a high-fat diet–induced obese mouse model (18). Although the impact of conventional soymilk on human lipid profiles remains inconsistent across studies, both human and murine experiments have consistently shown its beneficial effects in reducing LDL-C and TC levels, particularly among individuals with diabetes (19, 20). Collectively, these findings position soymilk as a functional food, providing a scientific basis for its application in the prevention and adjunctive therapy of metabolic disorders-including hyperlipidemia and diabetes.

Hyperlipidemia and the gut microbiota engage in bidirectional crosstalk, with mounting evidence supporting their reciprocal modulation and regulatory interplay (21, 22). Mouse studies by Tao et al. and Zheng et al. have demonstrated that reshaping the gut microbiota can modulate lipid metabolism and cholesterol homeostasis, consequently reducing circulating lipid concentrations (23, 24). Gut flora influences cholesterol metabolism, fat storage, and lipid peroxidation by participating in processes such as fatty acid absorption, bile acid metabolism, and fat storage. Consequently, it affects blood lipid levels (25). Kim et al. demonstrated in a high-fat diet (HFD)-induced mouse model that HFD profoundly disrupts the composition and function of the gut microbiota, leading to the overgrowth of pathogenic bacteria and the depletion of beneficial probiotics; such microbial dysbiosis further triggers disordered cholesterol metabolism by enhancing cholesterol absorption and synthesis, thereby aggravating hyperlipidemia (26). Research suggests that dietary adjustments, increased intake of dietary fiber, and the consumption of probiotics or prebiotics can improve the composition and function of intestinal flora (27). Howard et al. demonstrated in a murine model that dietary fiber intervention decreased the abundance of pathogenic gut microbiota while enriching beneficial bacteria, thereby regulating host lipid metabolic pathways and reducing circulating lipid concentrations (28). In summary, there exists a complex interaction between gut flora and hyperlipidemia. Maintaining a healthy gut flora can potentially contribute to alleviating hyperlipidemia. However, further research is needed to fully understand the specific mechanisms and develop effective therapeutic strategies.

Tea consumption also affects the intestinal flora. Rich in bioactive polyphenols and caffeine, tea-derived compounds reach the intestinal lumen during digestion and modulate the composition and function of the resident gut microbiota (29). Studies conducted in murine models have consistently demonstrated that the polyphenolic compounds in tea serve as a nutrient source for intestinal microorganisms, promoting the growth of beneficial flora (30, 31). Furthermore, tea polyphenols exert antimicrobial and anti-inflammatory effects, effectively attenuating high-fat diet–induced pathogenic bacterial proliferation in mice and thereby preserving gut microbiota homeostasis (32). Additionally, tea catechins exert potent antioxidant and anti-inflammatory effects, thereby mitigating high-fat diet–induced oxidative stress in the murine gut and preserving the compositional diversity of the intestinal microbiota (33). Soymilk modulates the gut microbiota mainly through fermentation-associated probiotic activity and compositional changes, and studies show that fermented soymilk increases the relative abundance of beneficial *Bifidobacterium* and *Lactobacillus* spp. while decreasing potential pathogens in mice (34). Furthermore, probiotics introduced during fermentation enhance antioxidant activity, inhibit the growth of detrimental bacteria in mice, and improve gut barrier function (35). However, unfermented soymilk or formulations with high protein content may elicit less significant shifts in microbiota composition, potentially due to differences in metabolic utilization (36). Collectively, the fermentation process and the addition of specific probiotics represent the key factors underlying soymilk’s capacity to beneficially modulate the gut microbiome, with effects potentially being more pronounced in individuals harboring equal-producing microbiota.

Kombucha soymilk is a beverage that combines the health benefits associated with soymilk and kombucha. Although kombucha, soymilk, and tea-derived bioactive compounds have each been reported to exert beneficial metabolic effects, evidence regarding kombucha-fermented soymilk as a combined functional intervention in diet-induced hyperlipidemia remains limited. In particular, it is still unclear whether kombucha soymilk is associated with coordinated alterations in gut microbial composition and host metabolic profiles, which may help explain its potential lipid-lowering effects. Therefore, in the present study, high-fat diet-induced obese animal models were employed as experimental subjects, and dietary intervention was conducted via oral gavage of kombucha soymilk. Body weight-related parameters, serum lipid levels, and hepatic antioxidant indices were detected, and liver pathological sections were observed to comprehensively and systematically evaluate the hypolipidemic potential of kombucha soymilk. Furthermore, 16S rRNA gene sequencing and untargeted metabolomic analysis were combined to systematically investigate the alterations in intestinal microbial and serum metabolic following the dietary intervention. This study attempts to provide preliminary scientific evidence for developing kombucha soymilk as a functional food for the prevention and intervention of hyperlipidemia, and seeks to lay a preliminary theoretical and experimental foundation for its potential application in the functional food field.

## 2 Materials and methods

### 2.1 Kombucha soymilk preparation

Kombucha Preparation: A mixed culture of *Saccharomyces cerevisiae* and *Gluconobacter oxydans* was prepared at a 1:1 (v/v) ratio. This inoculum was added to a green tea–sugar–water medium (green tea: sugar: water = 1: 10: 100, w/w) at a final inoculation volume of 10% (v/v). Fermentation was carried out under static conditions at 27 °C for 10 days to produce the green tea kombucha. Upon completion of fermentation, the green tea kombucha exhibited a final pH of 2.1, a residual soluble solids content (°Bx) of 2.5, an ethanol concentration of 1.8%, an acetic acid concentration of 7.50 g/L, a lactic acid concentration of 1.22 g/L, a glucuronic acid concentration of 2.36 g/L, and a viable cell count of 7.5 × 10^6^ CFU/mL.

Kombucha soymilk Preparation: Soak the soybeans in water for 16 h (soybeans: water = 1:10), then use a blender to make soy milk (4,000 r/min, fine grinding for 30 s, coarse grinding for 120 s, pause for 10 s, repeat fine grinding 5 times). Add 6% white granulated sugar to the soy milk and sterilize it at 121 °C for 20 min, then cool it for later use. Use green tea kombucha as the fermentation agent and inoculate it into the soy milk at a 10% inoculation rate. Ferment it at 28 °C for 30 h, then store it in a 4 °C refrigerator for 12 h to mature, and the kombucha soymilk is obtained.

### 2.2 Animals and diets

Specific pathogen-free (SPF) grade KM mice (male, 4-week-old, *n* = 18, initial body weight 22.00 ± 2.0 g) were purchased from Liaoning Changsheng Biotechnology Co., Ltd. (Production License: SCXK (Liao) 2020–0001). All animal procedures were approved by the Institutional Animal Care and Use Committee (IACUC) of Guangzhou Sihe Biotechnology Co., Ltd. (Number 2025-03-15-03). Mice were housed under standard conditions with ad libitum access to food and water, in a noise-controlled environment. The housing room was maintained at a constant temperature of 20 ± 2 °C and relative humidity of 55 ± 5%. The mice were provided with free access to water, and their dietary intake was monitored.

### 2.3 Pre-treatment, grouping, and diet regimen

Following a 1-week acclimation period, during which all mice were maintained on standard chow under thermoneutral conditions (24 ± 2 °C), the animals were randomly assigned to three groups (*n* = 6 per group):

B1. Control Group (Control): Fed standard chow diet.
B2. High-fat diet Group (HFD): Fed the high-fat diet.
B5. Treatment Group (HFD + KSM): Fed the high-fat diet plus oral gavage intervention with kombucha soybean milk.

Standard Chow Diet: Purchased from Ke’ao Xielì (Tianjin) Feed Co., Ltd. The diet consists of corn, soybean meal, fish meal, wheat flour, yeast powder, vegetable oil, sodium chloride, and a premix of vitamins and minerals. Its macronutrient energy distribution is as follows: protein 23.07%; fat 11.85%; carbohydrates 65.08%; total energy 3.40 kcal/g. This diet complies with the nutritional requirements specified in the People’s Republic of China National Standard GB 14924.3–2010, “Nutritional Components of Compound Feeds for Laboratory Animals.”

High Fat Diet (HFD): Purchased from Ke’ao Xielì (Tianjin) Feed Co., Ltd. Prepared by supplementing a standard chow diet with15% (w/w) lard, 20% (w/w) sucrose, 1.2% (w/w) cholesterol, and 0.2% (w/w) sodium cholate; fat-derived energy accounts for >36% of total caloric content.

### 2.4 Establishment of obese mouse models and dietary intervention protocol

After 8 weeks of high-fat diet induction, the mean body weight of mice in the Normal Control (NC) group was (43.61 ± 1.20) g per mouse, whereas that of mice in the High-fat Model (HM) group was (53.47 ± 1.50) g per mouse. The HM group exhibited a significantly higher body weight compared with the NC group, with an increase of 22.61%-exceeding the conventional 20% threshold used to confirm successful induction of the obesity model. Serum TC levels in the NC group were (2.42 ± 0.0153) mmol/L, and TG levels were (0.25 ± 0.0200) mmol/L; in contrast, TC and TG levels in the HM group were (3.92 ± 0.1756) mmol/L and (0.76 ± 0.0208) mmol/L, respectively. Compared with the NC group, both TC and TG levels in the HM group were significantly elevated (*p* < 0.01), indicating statistically significant differences. Therefore, a HFD-induced obese mouse model was successfully established. Body weight, TG, and TC levels measured at the time of model establishment were used as baseline values. Following successful induction of obesity, HFD-fed mice were further randomly divided into two groups: a high-fat control group and a kombucha soymilk intervention group (*n* = 6 per group).

Oral gavage administration was conducted daily throughout the 8-week experimental period:

B1. Control Group: Received daily gavage of physiological saline (500 μL/mouse).
B2. HFD Group: Received daily gavage of physiological saline (500 μL/mouse).
B3. HFD + KTSD Group: Received daily gavage of kombucha soybean milk (500 μL/mouse).

All gavage volumes were standardized to 500 μL per mouse. Investigators involved in sample handling, biochemical assessment, and histological evaluation were blinded to group allocation where applicable.

### 2.5 Sample collection and processing

At the end of the eighth week, mice were fasted overnight (12 h) with continued access to water. Blood samples were collected via retro-orbital bleeding under appropriate anesthesia, followed immediately by euthanasia via cervical dislocation. Fresh blood was centrifuged (4 °C, 3,500 rpm, 10 min) to separate serum and plasma. Serum aliquots were stored at −80 °C for subsequent analysis. Hearts, livers, and epididymal fat pads were rapidly excised and weighed.

### 2.6 Liver tissue processing

Liver tissues designated for histological analysis were immediately fixed in 4% paraformaldehyde, followed by dehydration and embedding in paraffin. Liver sections (5 μm thickness—Note: corrected from 100 μm; 100 μm is atypical for H&E/Oil Red O) were prepared for Hematoxylin and Eosin (H&E) and Oil Red O staining. The remaining liver tissue aliquots were snap-frozen and stored at −80 °C.

### 2.7 Biochemical analysis

Total cholesterol (TC), triglycerides (TG), low-density lipoprotein cholesterol (LDL-C), high-density lipoprotein cholesterol (HDL-C), glutathione peroxidase (GSH-Px), superoxide dismutase (SOD), and malondialdehyde (MDA) were quantified using commercial enzymatic assay kits (Suzhou Michy Biomedical Technology Co., Ltd., China) according to the manufacturer’s instructions.

### 2.8 Fecal microbiota analysis by 16S rRNA gene sequencing

Fresh fecal samples were collected aseptically from individual mice at the end of the intervention and immediately stored at −80 °C until analysis. Total microbial DNA was extracted using a cetyltrimethylammonium bromide (CTAB)-based protocol. Briefly, fecal samples were lysed in preheated CTAB extraction buffer at 65 °C, followed by chloroform-isoamyl alcohol extraction, isopropanol precipitation, ethanol washing, and RNase treatment. DNA integrity was evaluated by agarose gel electrophoresis, and DNA concentration and purity were assessed spectrophotometrically.

The V3–V4 hypervariable region of the bacterial 16S rRNA gene was amplified using the universal primers 341F (5′-CCTACGGGNGGCWGCAG-3′) and 805R (5′-GACTACHVGGGTATCTAATCC-3′). PCR amplification was performed in a 25 μL reaction system containing 12.5 μL Phusion Hot Start Flex 2 × Master Mix, 2.5 μL of each primer, 50 ng template DNA, and nuclease-free water to volume. The thermocycling conditions were as follows: initial denaturation at 98 °C for 30 s; 32 cycles of 98 °C for 10 s, 54 °C for 30 s, and 72 °C for 45 s; followed by a final extension at 72 °C for 10 min. Amplicons were verified by 2% agarose gel electrophoresis, purified using AMPure XT beads, quantified with Qubit, and assessed for library quality using an Agilent 2100 Bioanalyzer.

Qualified libraries were pooled and sequenced on the Illumina NovaSeq 6000 platform using 2 × 250 bp paired-end chemistry. Raw reads were demultiplexed according to barcode sequences. Primer and adaptor sequences were removed using cutadapt (v1.9), paired-end reads were merged using FLASH (v1.2.8), low-quality reads were filtered using fqtrim (v0.94), and chimeric sequences were removed using Vsearch (v2.3.4). Sequence denoising was performed in QIIME2 (2019.7) with the DADA2 plugin to generate amplicon sequence variants (ASVs). Singleton ASVs and very low-abundance features were removed prior to downstream exploratory analyses to reduce noise. Taxonomic assignment was performed against the SILVA database (release 138) with a confidence threshold of 0.7.

Alpha-diversity indices, including Observed species, Chao1, Shannon, Simpson, Goods coverage, and Pielou’s evenness, were calculated to evaluate within-sample richness and diversity. Beta diversity was assessed using weighted UniFrac, unweighted UniFrac, Jaccard, and Bray-Curtis distance matrices and visualized by PCA, PCoA, NMDS, and UPGMA clustering. Differential taxonomic comparisons were performed at the genus level under an exploratory framework.

### 2.9 Serum metabolomics analysis

Serum samples were subjected to untargeted metabolomics analysis using a commercial liquid chromatography-mass spectrometry (LC–MS)-based platform. Frozen serum samples were thawed on ice and processed by protein precipitation and metabolite extraction using organic solvent according to the platform’s standard operating procedure. After centrifugation, the supernatants were collected for instrumental analysis. Pooled quality-control (QC) samples were prepared by mixing equal aliquots from representative serum samples and were analyzed intermittently throughout the analytical run to monitor instrument stability and data reproducibility.

Raw LC–MS data were processed using the provider’s standard workflow, including peak detection, deconvolution, alignment, retention-time correction, and normalization. Metabolite annotation was performed by matching accurate mass, retention behavior, and, where available, MS/MS fragmentation information against commercial and public spectral databases used by the analytical platform. Because this was an untargeted metabolomics study performed through a commercial service platform, metabolite identities should be considered putative unless otherwise confirmed by authentic reference standards.

Multivariate analyses, including principal component analysis (PCA), were used for an overview of the metabolic profiles among groups. Differential metabolites were screened using the platform’s integrated statistical workflow based on fold change and significance testing, and false discovery rate (FDR) adjustment was applied where appropriate for multiple comparisons in exploratory analyses. KEGG pathway enrichment analysis was subsequently performed to facilitate biological interpretation of the altered metabolic features.

To avoid overinterpretation, metabolomics findings in the present study were treated as exploratory associations. Direct attribution of individual metabolites to specific tea- or soy-derived bioactive compounds was not considered definitive in the absence of targeted validation.

### 2.10 Statistical analysis

Statistical analyses were performed using SPSS software. All data were expressed as mean ± standard deviation (SD). Statistical comparisons between two groups or pairwise comparisons among three groups were performed using independent-sample Student’s *t*-test, with ** or ## representing highly significant differences (*p* < 0.01), and with * or # representing significant differences (*p* < 0.05). For comparisons among more than three groups, one-way analysis of variance (ANOVA) followed by *post hoc* tests was conducted. Different lowercase letters indicate significant differences (*p* < 0.05), whereas different uppercase letters indicate highly significant differences (*p* < 0.01). *p* < 0.05 was considered statistically significant.

For microbiome analysis, alpha-diversity indices were compared using non-parametric tests as appropriate. Beta-diversity differences were evaluated by PERMANOVA (Adonis) based on distance matrices. Relative abundances of selected taxa were compared using the Mann–Whitney U test for pairwise analyses or the Kruskal-Wallis test for three-group comparisons. Because microbiome and metabolomics datasets are high-dimensional and exploratory in nature, *p* values from multiple comparisons were adjusted using the Benjamini-Hochberg false discovery rate (FDR) method where applicable. Taxa or metabolites with nominal significance but without FDR support were interpreted cautiously.

For metabolomics analysis, multivariate exploratory analyses were combined with univariate screening and pathway enrichment. Because of the limited sample size and exploratory design, all microbiome- and metabolome-related findings were interpreted as associative rather than causal.

## 3 Results

### 3.1 The consumption of kombucha soymilk has been shown to reduce and alleviate symptoms of hyperlipidemia in mice

**Figure 1:**
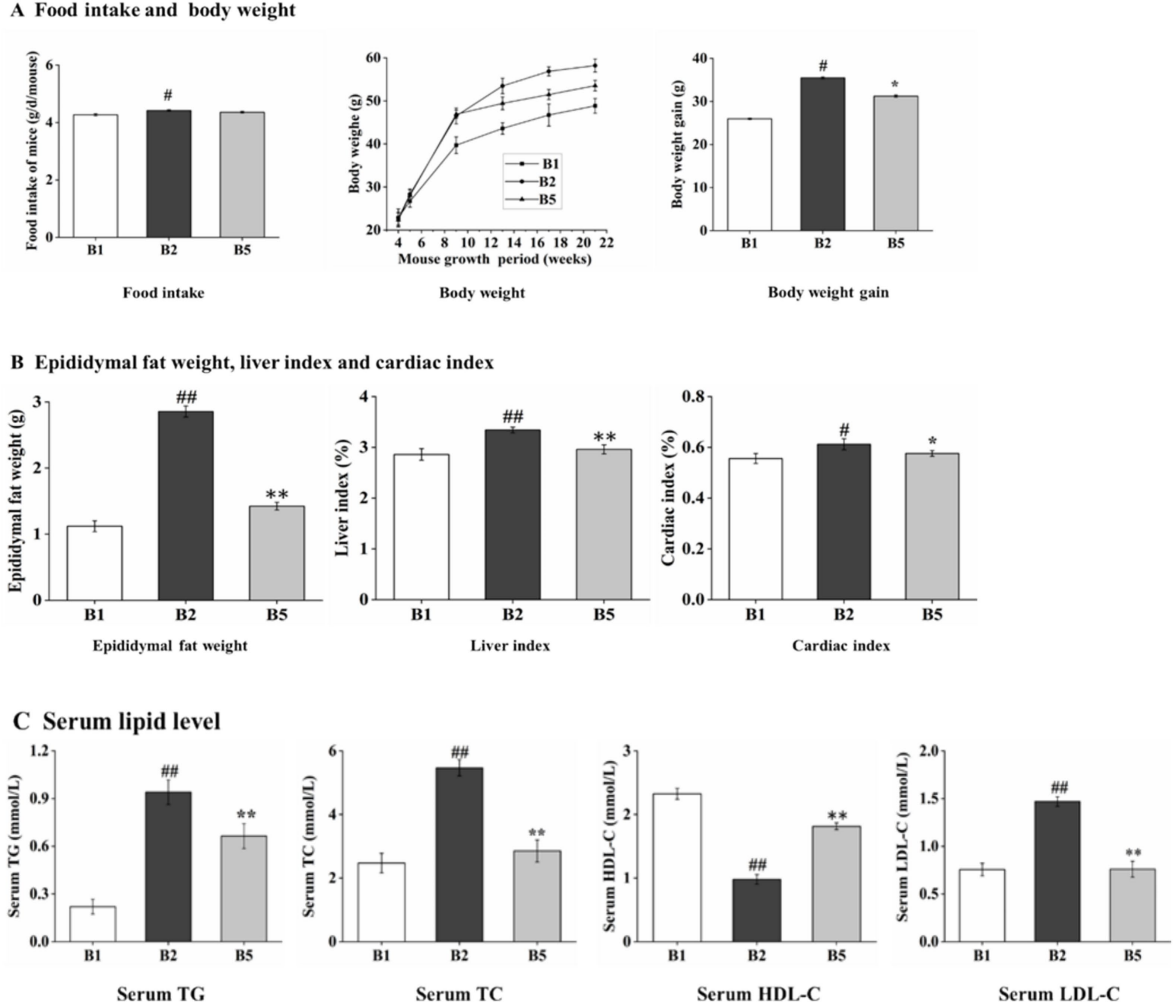
Effects of kombucha soymilk on food intake, weight parameters and serum lipid level in high fat mice. **(A)** Food intake, body weight, body weight gain. **(B)** Epididymal fat weight, liver index, cardiac index. **(C)** Serum lipid level (TG, TC, HDL-C, LDL-C). Data are presented as mean ± SD. Independent-sample *t*-test was used for statistical analysis. #*p* < 0.05 B2 versus B1, *p < 0.05 B2 versus B5; ##*p* < 0.01 B2 versus B1, and **p < 0.01 B2 versus B5.

As shown in Figure 1A, no significant difference in food intake was observed between the B5 (4.36 ± 0.0321 g/d/mouse) and B2 (4.42 ± 0.0251 g/d/mouse) groups, suggesting that kombucha soymilk dietary intervention had no effect on appetite in mice; however, food intake differed significantly between the B2 and B1 (4.27 ± 0.0378 g/d/mouse) groups, which may be attributable to the higher palatability of the high-fat diet fed to the B2 group, thereby promoting increased appetite. We observed that the body weight gain of the B5 group (31.23 ± 0.2517 g) was significantly lower than that in the B2 group (35.48 ± 0.2359 g) (*p* < 0.05), indicating that kombucha soymilk dietary intervention effectively suppressed body weight gain in mice; moreover, body weight gain of the B2 group was significantly higher than that of the B1group (25.97 ± 0.1528 g) (*p* < 0.05), suggesting that prolonged high-fat diet intake leads to sustained body weight increase.

According to our study conducted on mice, it has been found that kombucha soymilk can effectively reduce and alleviate symptoms of hyperlipidemia. As shown in Figure 1B, the B2 group exhibited a significantly higher epididymal fat weight (2.86 ± 0.0839 g) compared with the B1 group (1.12 ± 0.0825 g) (*p* < 0.01), whereas the B5 group (1.42 ± 0.0581 g) exhibited a significantly lower value relative to the B2 group (*p* < 0.01). Similarly, the liver index of the B2 group (3.34 ± 0.0548) was significantly elevated compared with the B1 group (2.86 ± 0.1140) (*p* < 0.01), and that of the B5 group (2.96 ± 0.0894) was significantly reduced relative to the B2 group (*p* < 0.01). In addition, the cardiac index was significantly higher in the B2 group (0.61 ± 0.0217) than in the B1 group (0.55 ± 0.0195) (*p* < 0.05), while the B5 group (0.58 ± 0.0114) showed a significantly lower cardiac index compared with the B2 group (*p* < 0.05). Notably, the B5 group showed a marked restoration in these parameters, indicating a significant recovery from the alterations observed in the B2 group.

This study analyzed the serum lipid profiles of three groups of mice. The results demonstrated that the B5 group exhibited significantly lower levels of TG (0.66 ± 0.0503 mmol/L), TC (2.85 ± 0.1323 mmol/L), and LDL-C (0.84 ± 0.0208 mmol/L) compared with the B2 group, with respective reductions of 47.88, 29.79, and 44.73% (*p* < 0.01). In contrast, the high-density lipoprotein HDL-C level of the B2 group was significantly higher than that of the B5 group (1.81 ± 0.0518 mmol/L), representing an increase of 84.69% (*p* < 0.01) (Figure 1C). However, it should be noted that the reduction in blood lipid levels in the B5 group did not reach the same level as B1 group. These findings suggest that kombucha soymilk consumption may have a positive impact on managing hyperlipidemia by regulating body weight and serum lipid levels health.

### 3.2 The consumption of kombucha soymilk improves liver biochemical parameters and histopathology in high fat diet mice

**Figure 2:**
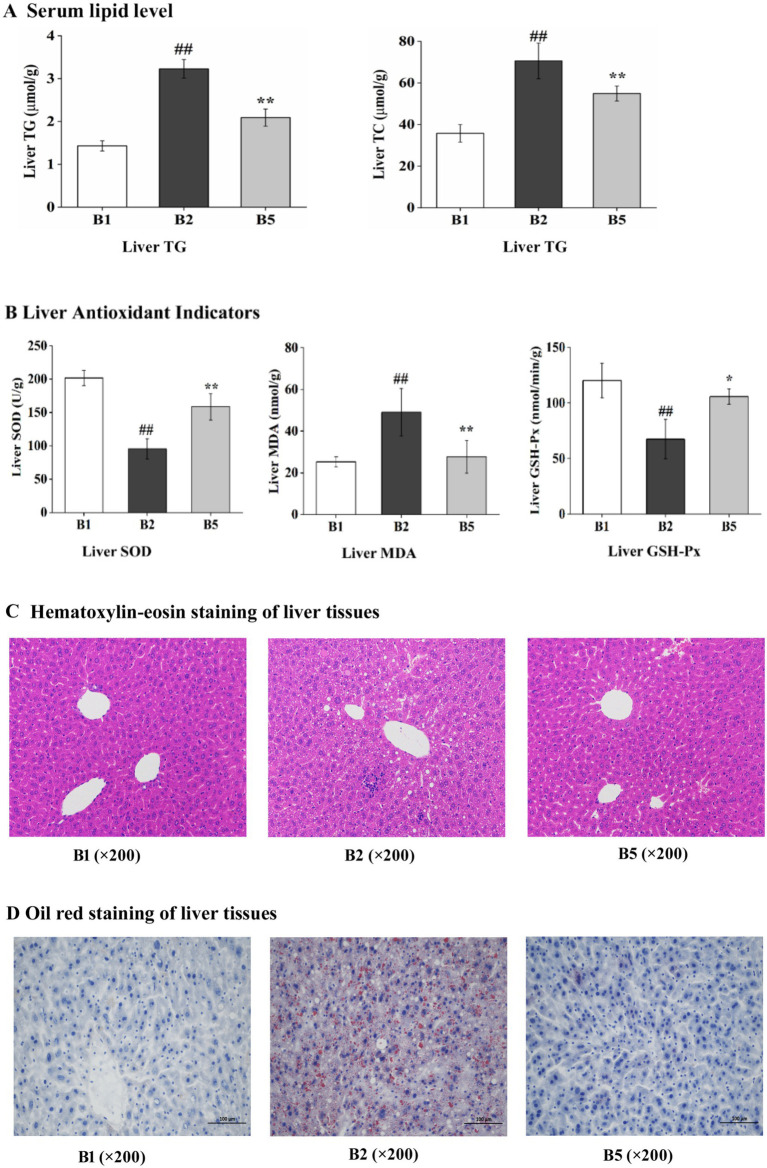
Effects of kombucha soymilk on the liver biochemical parameters and histopathology in high fat mice. **(A)** Liver lipid level (TG, TC). **(B)** Liver SOD, liver MDA, liver GSH-Px. **(C)** Hematoxylin–eosin staining of liver tissues. **(D)** Oil red staining of liver tissues. Data are presented as mean ± SD. Independent-sample t-test was used for statistical analysis. **(C,D)** Representative histological images. #*p* < 0.05 B2 versus B1, *p < 0.05 B2 versus B5; ##*p* < 0.01 B2 versus B1, and **p < 0.01 B2 versus B5.

This study investigated the effects of kombucha soymilk on liver health. Specifically, our experiments conducted on mice demonstrated that kombucha soymilk could effectively improve hepatic lipid levels. Compared with the B2 group, the B5 group exhibited significant decreases in hepatic TG (2.09 ± 0.1992 μmol/g) and TC (54.92 ± 3.6403 μmol/g), with respective reductions of 35.29 and 22.21% (*p* < 0.01) (Figure 2A). We analyzed hepatic oxidative stress status in three groups of mice. Specifically, in the B5 group, significantly elevated levels of superoxide dismutase (SOD, 158.41 ± 19.7216 U/g) and glutathione peroxidase (GSH-Px, 105.77 ± 6.8469 nmol/min/g) were observed compared with the B2 group, with respective increases of 65.99 and 56.60% (*p* < 0.05). Additionally, the malondialdehyde (MDA) level in the B5 group was significantly reduced by 43.50% relative to that in the B2 group (*p* < 0.01) (Figure 2B). The histological analysis of liver showed that kombucha soymilk could reduce hepatocyte damage in mice. The results of hematoxylin–eosin staining of liver tissues indicated that the liver tissues in the B1 group were closely arranged and exhibited no signs of degeneration. In contrast, the B2 group displayed lesions characterized by white fat vacuoles interspersed among the tissue. Notable improvements were observed in the B5 group, with histological morphology demonstrating significant recovery (Figure 2C). The results of Oil Red staining revealed that the B1 group exhibited no discernible lipid droplets. In contrast, the B2 group displayed relatively distinct lipid droplets. Notably, the B5 groups demonstrated significant improvement, characterized by a markedly reduced presence of lipid droplets (Figure 2D).

These findings indicate that the consumption of kombucha soymilk may positively influence the management of hyperlipidemia by alleviating hepatic injury. However, further research is still needed to fully understand the mechanisms and potential benefits of kombucha soymilk in combating this condition.

### 3.3 General characteristics of intestinal flora in hyperlipidemia mice with the consumption of kombucha soymilk

**Figure 3:**
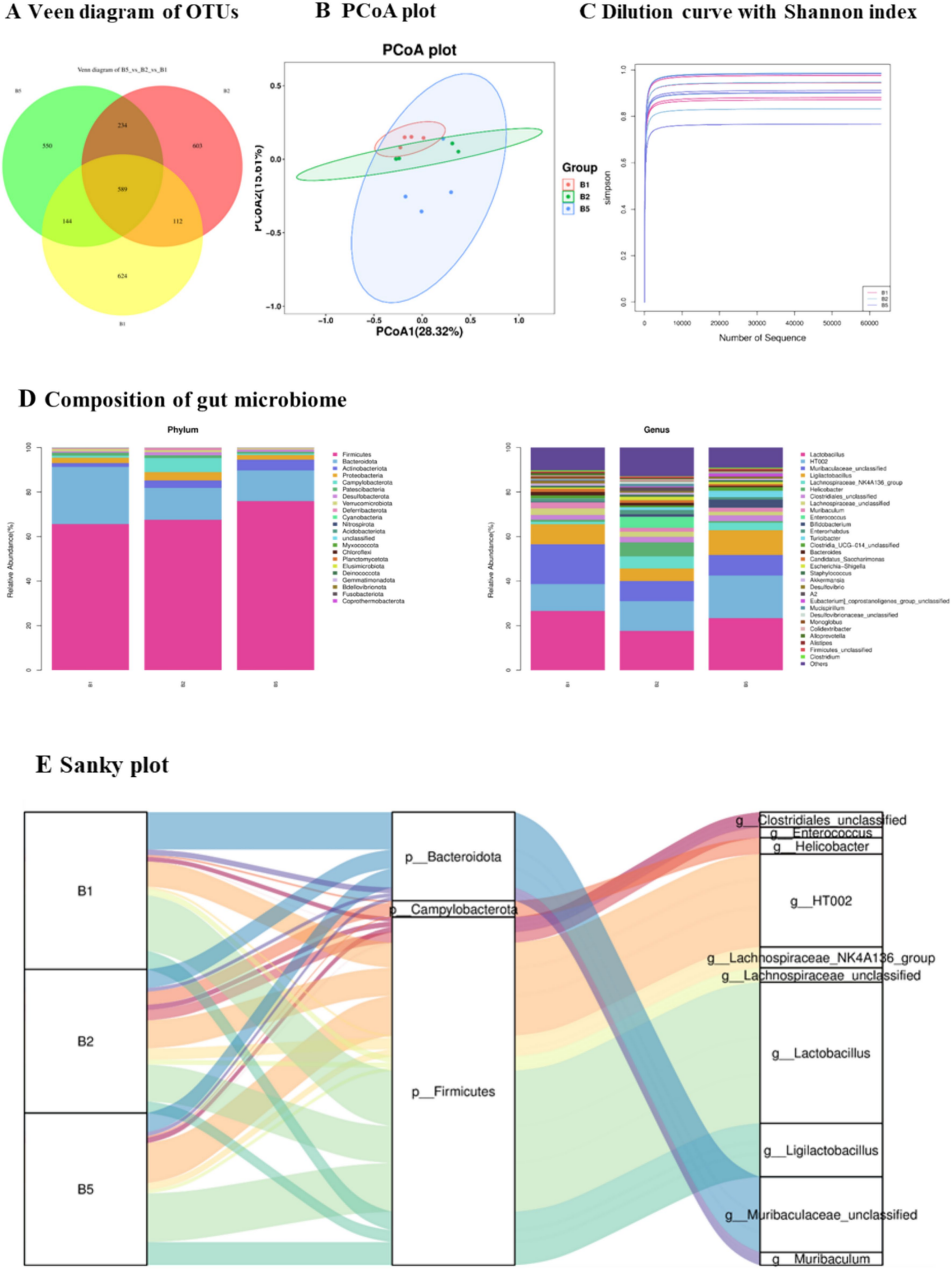
General characteristics of the gut microbiome in hyperlipidemia mice receiving kombucha soymilk. **(A)** Venn diagram of ASVs in the control (B1), HFD (B2), and kombucha soymilk-treated (B5) groups. **(B)** PCoA plot of beta diversity; group differences were evaluated by PERMANOVA (Adonis) based on distance matrices. **(C)** Alpha-diversity indices, including Shannon and Chao1, were compared using the Kruskal-Wallis test. **(D)** Composition of gut microbiome in phylum and genus levels. **(E)** Sanky plot.

In this study focusing on the intestinal microbiota, the bacterial 16S rRNA gene V3-V4 region was analyzed in fecal samples from the three experimental groups. After denoising and quality control, amplicon sequence variants (ASVs) were obtained for subsequent taxonomic annotation and diversity analyses (Figure 3A). Alpha-diversity indices, including Shannon and Chao1, were used to evaluate within-sample richness and diversity (Figure 3B).

Principal coordinate analysis (PCoA) was used to visualize differences in community structure among groups. Although clear global clustering separation was not observed among the three groups (Figure 3B), local trends in beta diversity were apparent, and subsequent taxonomic analyses suggested that kombucha soymilk intake was associated with shifts in specific bacterial taxa rather than a strong overall restructuring of the gut microbiota.

To further characterize the taxonomic distribution of microbial communities, the numbers of corresponding ASVs were summarized and visualized at the phylum and genus levels (Figures 3D,E). These analyses provided an overview of community composition in each group and supported subsequent differential-abundance analyses.

These findings provide valuable insights into the composition and diversity of intestinal flora within different groups and highlight potential differences in community structure and taxonomic distribution. Further studies are warranted to explore the functional implications of these findings and their relevance to human health.

### 3.4 Bacteria with distinct abundance patterns in hyperlipidemia mice with the consumption of kombucha soymilk

To examine taxonomic differences among groups, the relative abundances of representative taxa were compared using non-parametric statistical methods. Pairwise comparisons were performed using the Mann–Whitney U test, whereas comparisons across all three groups were performed using the Kruskal-Wallis test. Differentially abundant taxa were interpreted cautiously because the microbiome analysis was exploratory and the sample size was limited.

**Figure 4:**
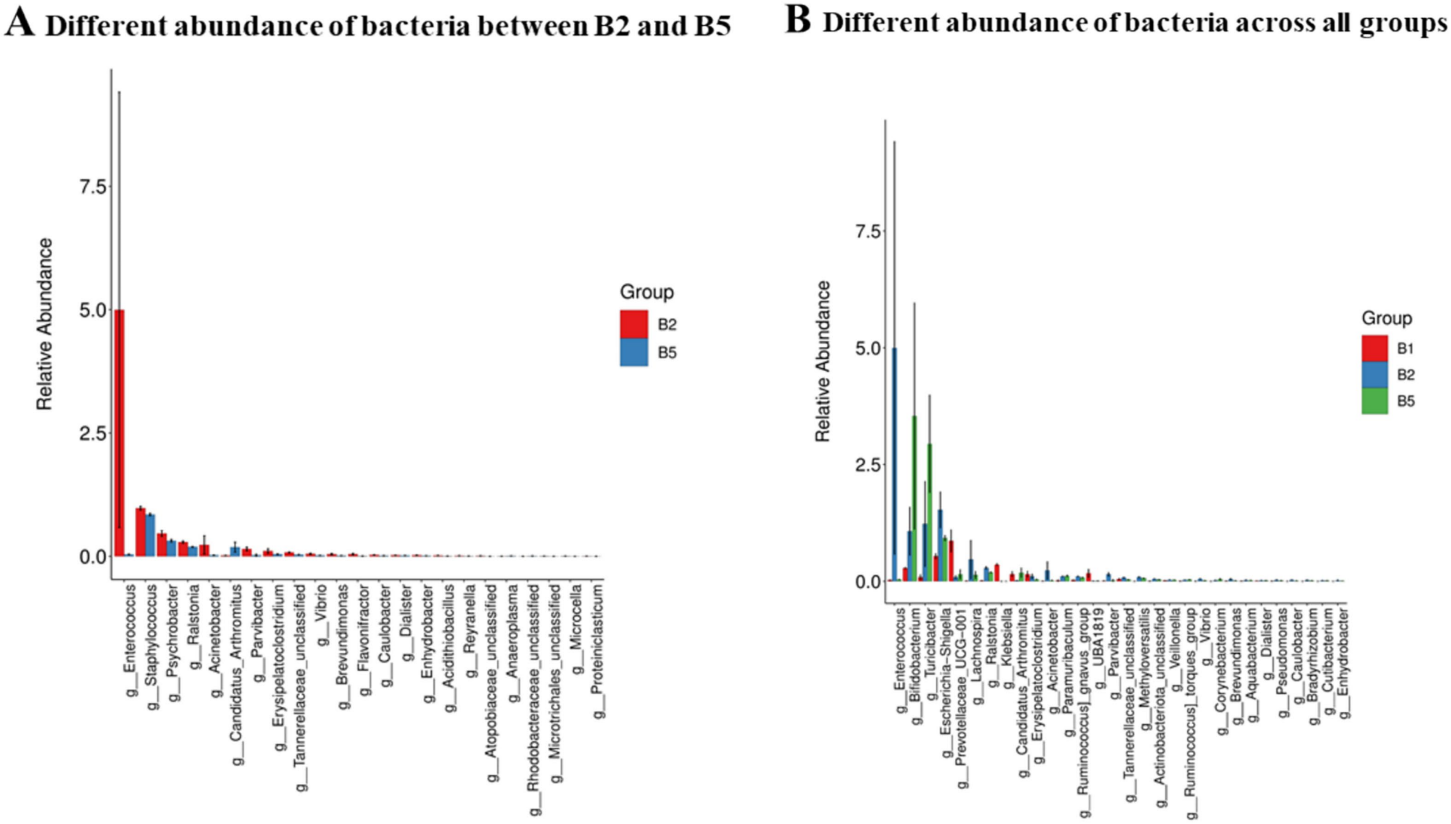
Differentially abundant bacteria among mouse groups. **(A)** Comparison between the HFD group (B2) and kombucha soymilk-treated group (B5). **(B)** Comparison across all three groups. Pairwise taxonomic comparisons were analyzed using the Mann–Whitney U test, and three-group comparisons were analyzed using the Kruskal-Wallis test. Biomarker discovery, where applicable, followed the LEfSe workflow.

In terms of the divergence between the control and kombucha soymilk groups, the most distinct genera were *Bifidobacterium* and *Turicibacter*. Additionally*, Enterococcus* (B2) was the genus that showed considerable differences between the HFD and kombucha soymilk groups. Among the three groups, the most significantly divergent genera were *Enterococcus (B2), Bifidobacterium,* and *Turicibacter* (Figure 4).

These findings highlight the specific bacterial species and genera that display notable variations in abundance across the different groups studied. Further investigations are warranted to explore the functional roles and potential implications of these bacterial differences in relation to the health outcomes associated with each group.

### 3.5 General metabolome information in hyperlipidemia mice with the consumption of kombucha soymilk

**Figure 5:**
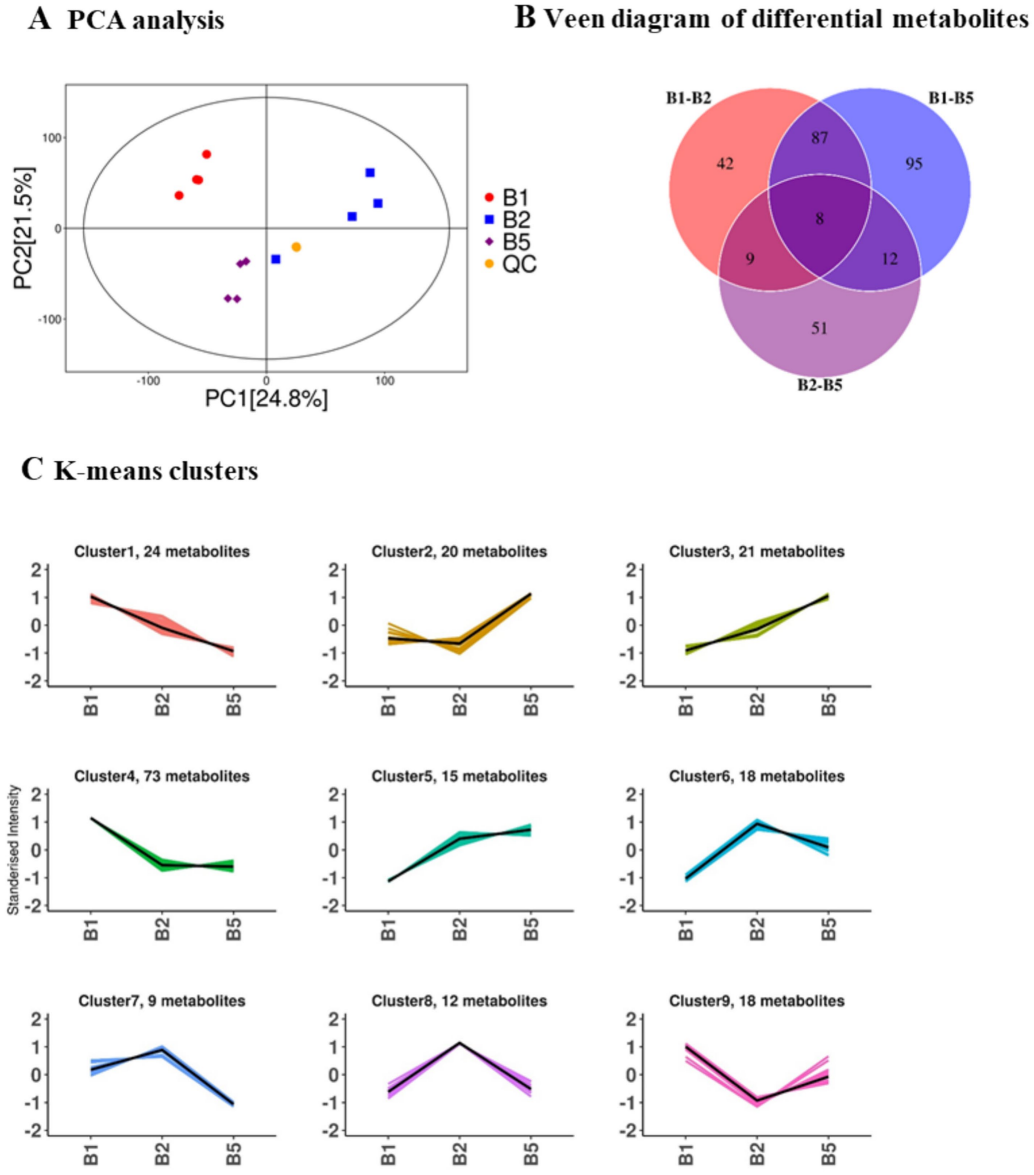
General metabolomic characteristics in hyperlipidemia mice receiving kombucha soymilk. **(A)** PCA analysis of serum metabolomic profiles. **(B)** Venn diagram of differential metabolites. **(C)** *K*-means clustering of metabolite abundance patterns. The metabolomics results were considered exploratory and were interpreted together with pathway enrichment analysis.

Principal component analysis (PCA) was performed to analyze the metabolome structure of the three subgroups, revealing significant differences as the samples resided in different quadrants (Figure 5A). Veene plots were used to visualize the distribution of differential metabolites among the groups (Figure 5B). The analysis identified 8 metabolites that were common to all three differential groups, 42 metabolites unique to the B1vsB2 comparison, 95 metabolites unique to the B1vsB5 comparison, and 51 metabolites unique to the B2vsB5 comparison.

Based on the metabolite abundance in each group, the metabolites were classified into 9 clusters using K-means clustering (Figure 5C). Cluster 8 contained 12 metabolites, which showed the highest abundance in the HFD group of B2. The kombucha soymilk group of B5 exhibited similar levels to the control group of B1 for these metabolites, including Dethiobiotin, DL-Dopa, H-GLU (ALA-OH)-OH, Alpha-dimorphecolic acid, 7-beta- Hydroxylathyrol, Febrifugine, Prolyl-Arginine, [8]-Gingerol, Ponicidin, Linoelaidyl carnitine, (3R)-3,4-Dihydroxy-3-(hydroxymethyl) butanenitrile 4-glucoside, and PA. Based on the described metabolites, the following are those related to tea polyphenols, soy isoflavones, soy saponins, and proteins. DL-Dopa, [8]-Gingerol and Ponicidin are related to similar compounds found in tea polyphenols. Alpha-dimorphecolic acid, Ponicidin and (3R)-3, 4-Dihydroxy-3-(hydroxymethyl) butanenitrile 4-glucoside are associated with the metabolic pathway of soy isoflavones. Linoelaidyl carnitine is a carnitine ester that may be related to fatty acid metabolism and soy saponins. Dethiobiotin, Febrifugine and Prolyl-Arginine are related to protein synthesis or metabolism. Therefore, these metabolites may be involved in the study’s correlation with tea polyphenols, soy isoflavones, soy saponins, and proteins.

Furthermore, cluster 9 comprised 18 metabolites with the lowest abundance in the HFD group of B2 and comparable levels in the kombucha soymilk group of B5 and the control group of B1. Some metabolites in this cluster included Pyrrolidine, N-Acetyl-L-alanine, Orotate, MeAIB, N-Acetyl-L-arginine, Amifampridine, Cholesterol, 5-Pyridoxolactone, N-Alpha-acetyllysine, beta-Cryptoxanthin, Sciadonic acid, Thiodiglycol, 12-HPETE, Docosahexaenoic acid, 1, 3, 7-Trimethyluric acid, (10E,12Z)-(9S)-9-Hydroperoxyoctadeca-10,12-dienoic acid, Glutathione, and Adipic acid. All the above metabolites are unrelated to tea polyphenols, soy isoflavones, soy saponins, and proteins.

Pathway enrichment analysis of the differential metabolites revealed their enrichment in pathways such as Bile secretion, Regulation of lipolysis in adipocytes, Steroid biosynthesis, and Linoleic acid metabolism.

These findings provide a glimpse into the composition and pathways associated with the metabolome, shedding light on potential metabolic differences and their functional implications across the studied groups. Further investigations are necessary to elucidate the precise roles of these metabolites and pathways in relation to the physiological and disease processes under investigation.

### 3.6 Differential metabolites in hyperlipidemia mice with the consumption of kombucha soymilk

When focusing on the differential metabolites between B2 and B5, the heat map analysis revealed that these metabolites could be divided into two main groups based on their higher abundance in either B2 or B5. Metabolites like N-Acetyl-L-arginine, among others, exhibited higher abundance in the B5 kombucha soymilk group, while metabolites like D-Lactic acid, among others, showed higher abundance in the B2 HFD group.

**Figure 6:**
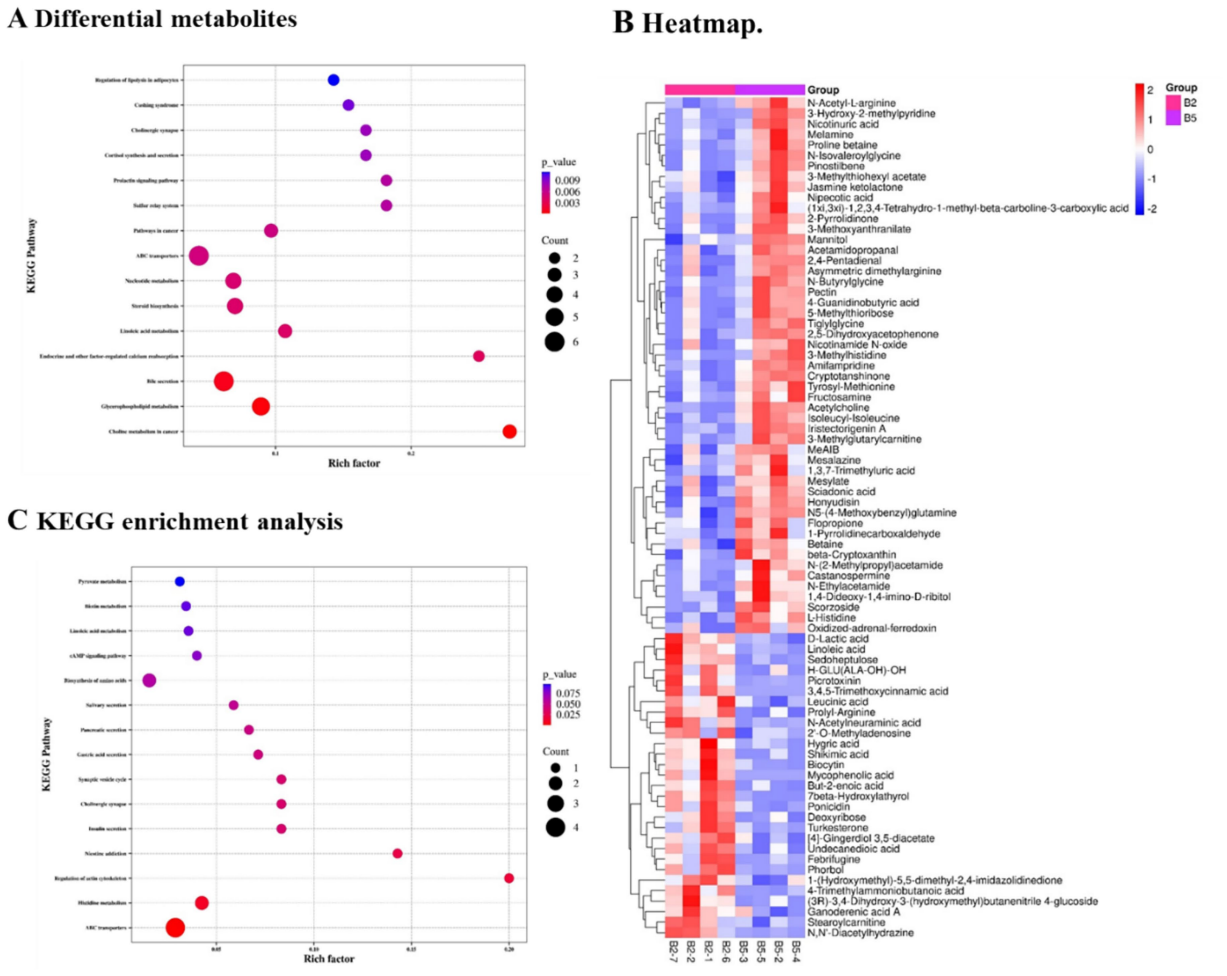
Differential metabolites between the HFD group (B2) and kombucha soymilk-treated group (B5). **(A)** KEGG enrichment analysis of differential metabolites. **(B)** Heatmap of representative differential metabolites. **(C)** Additional KEGG enrichment analysis. Correlation analyses, where applicable, were performed using Spearman correlation.

To gain further insights into the functional implications of these differential metabolites, KEGG enrichment analysis was conducted (Figure 6A). The results indicated that these metabolites were primarily enriched in pathways such as cAMP signaling pathway, Tyruvate metabolism, Linoleic acid metabolism, and other relevant pathways.

The heat map analysis provides a visual representation of the distinct abundance patterns of metabolites between the B2 and B5 groups (Figure 6B). Furthermore, the KEGG enrichment analysis offers valuable information regarding the potential biological pathways associated with these differential metabolites (Figure 6C). Further investigations are needed to decipher the precise roles of these metabolites and their related pathways in the context of the B2 and B5 groups, shedding light on their functional significance in the studied conditions.

## 4 Discussion

Kombucha soymilk has shown promising potential in positively influencing hyperlipidemia by modulating gut flora. Gut flora, which refers to the diverse community of microorganisms residing in the human intestinal tract, plays a crucial role in maintaining overall health and supporting immune system function. Emerging research suggests that certain components of kombucha soymilk, such as catechins and caffeine, have the ability to shape the composition of gut flora. These kombucha soymilk components, including catechins and polyphenolic compounds, contribute to the alteration of intestinal flora, promoting the growth of beneficial bacteria while inhibiting the proliferation of harmful bacteria. Hyperlipidemia, characterized by elevated levels of cholesterol and fat in the body, can potentially be addressed by the lipid-lowering properties of kombucha soymilk. Studies have demonstrated that the bioactive compounds in kombucha soymilk can help reduce blood lipids. By improving the balance of intestinal flora, kombucha soymilk may effectively reduce blood cholesterol and fat levels, thus diminishing the risk of developing hyperlipidemia. Despite these promising findings, further investigations are warranted to fully comprehend the intricate mechanisms underlying the relationship between kombucha soymilk and gut flora, as well as to optimize the potential benefits of kombucha soymilk in managing hyperlipidemia.

This study has documented notable differences in bacterial populations between the groups receiving kombucha soymilk and those in the control group, which have been reported to be associated with hyperlipidemia. These findings provide valuable insights into the potential impact of kombucha soymilk on gut flora and its relevance to hyperlipidemia management. Several studies have demonstrated the potential of different strains of *Enterococcus faecium* and *Enterococcus faecalis*, as well as *Bacillus subtilis*, to prevent obesity-associated hyperlipidemia and modulate the composition of gut microbiota in animal models. In one study, the combination of *Enterococcus faecium* R0026 and *Bacillus subtilis* R0179 was found to effectively prevent obesity-related hyperlipidemia and modify gut microbiota in C57BL/6 mice (37). Another study focused on *Enterococcus faecium* WEFA23 and demonstrated its ability to reduce high-fat-diet-induced hyperlipidemia in rats by targeting the cholesterol 7-alpha-hydroxylase gene and influencing gut microbiota composition (38). Additionally, *Enterococcus faecalis* was found to have beneficial effects on cholesterol transportation and gut microbiota in hypercholesterolemic mice (39). Furthermore, the cholesterol-lowering mechanisms of *Enterococcus faecium* strain 132 and *Lactobacillus paracasei* strain 201 were evaluated in hypercholesterolemia rats (40). Research has consistently highlighted *Bifidobacterium* as a common and predominant probiotic found in the intestinal tract. Studies have demonstrated a positive correlation between the remission of hyperlipidemia and an increase in the abundance of *Bifidobacterium* (41). Additionally, *Turicibacter* and Lactobacillus have also been identified as typical and abundant probiotic organisms within the gut. These findings underscore the potential role of these specific probiotic strains in managing hyperlipidemia. The presence and proliferation of *Bifidobacterium*, *Turicibacter*, and Lactobacillus may contribute to the modulation of gut flora and the improvement of lipid profiles (42, 43). Further research is needed to elucidate the precise mechanisms through which these probiotics exert their beneficial effects on hyperlipidemia and to explore their potential as therapeutic interventions. These studies collectively contribute to our understanding of the potential therapeutic applications of specific bacterial strains in managing hyperlipidemia and modulating gut microbiota composition.

Our research conducted on different groups of mice has revealed significant variations between the groups, which have distinct impacts on mouse metabolism. One of the prominent differences lies in the metabolites associated with steroid biosynthesis and succinic acid. Steroid biosynthesis reflects the process of synthesizing and metabolizing steroid hormones within the body. Analysis has demonstrated marked differences in the metabolic pathways of steroid biosynthesis among the various groups of mice. This suggests that the synthesis and metabolism of steroids may undergo alterations in mice subjected to different treatment conditions. Furthermore, substantial differences have been observed in the levels of succinic acid, an important intermediate metabolite involved in energy metabolism and oxidative pathways. These variations hint at potential changes in energy metabolism and oxidative pathways across the different mouse groups. The identification of these discrepancies provides valuable insights for comprehending the specific effects of distinct treatments on *in vivo* metabolism in mice. Further exploration of these differences shall facilitate a deeper understanding of the impacts on metabolism, thus serving as valuable references for future research and therapeutic approaches.

According to our research, metabolites such as DL-Dopa, [8]-Gingerol, and Ponicidin are related to compounds found in tea polyphenols. Additionally, Alpha-dimorphecolic acid, Ponicidin, and (3R)-3,4-Dihydroxy-3-(hydroxymethyl) butanenitrile 4-glucoside are associated with the metabolic pathways of soy isoflavones. Linoelaidyl carnitine is a creatine ester potentially linked to fatty acid metabolism and soy saponins. Furthermore, Dethiobiotin, Febrifugine, and Prolyl-Arginine are involved in protein synthesis or metabolism. We observed elevated levels of these metabolites in the B2 HFD group, whereas their levels normalized in the B5 kombucha soymilk-drinking group. These metabolites are key components of kombucha soymilk, such as polyphenols, soy isoflavones, soy saponins, and proteins. Our findings suggest that the HFD group accelerates the metabolism of these nutrients, whereas kombucha soymilk consumption slows their metabolism.

From a scientific perspective, kombucha soymilk consumption may contribute to metabolic regulation by modulating gut microbial composition and metabolite profiles. However, the present study did not directly demonstrate slowed metabolism, enhanced bioavailability, or causal mechanisms for individual compounds; therefore, these interpretations should be regarded as hypothesis-generating rather than definitive.

This study demonstrated that after 8 weeks of kombucha soymilk intervention, serum TG and TC levels in mice of the kombucha soymilk group (B5) were significantly lower than those in the model group (B2) (*p <* 0.01) (Supplementary Table S1), suggesting that regular and continuous kombucha soymilk supplementation effectively improves blood lipid levels in high-fat diet-induced obese mice. Following successful establishment of the obese mouse model, serum TG and TC levels in the blank control and model groups were determined prior to kombucha soymilk intervention to obtain baseline values. Compared with the baseline levels at the completion of modeling, TG and TC levels were significantly elevated in the model group (B2) (*p <* 0.01). In the intervention group (B5), serum TG and TC were significantly lower than the baseline levels (*p <* 0.01) (Supplementary Table S2). These results suggest that kombucha soymilk intervention may effectively attenuate the increase in TG and significantly inhibit the further elevation of TC induced by high-fat diet feeding, thus contributing to an improved lipid level and exerting a beneficial effect against dyslipidemia. Meanwhile, monitoring of food intake showed no significant difference between the kombucha soymilk intervention group (B5) and the model group (B2), suggesting that the differences in lipid levels between the two groups could not be explained by variations in energy intake. Accordingly, the lipid-lowering effect of kombucha soymilk intervention may be attributed to its regulatory effects on the gut microbiota structure, modulation of systemic metabolite, and subsequent regulation of lipid metabolism pathways.

In conclusion, although the overall beta-diversity differences among groups were not statistically robust, specific microbial taxa and serum metabolites were associated with kombucha soymilk intervention. These findings suggest a potential link between kombucha soymilk intake, gut microbiota modulation, and metabolic improvement in high-fat diet mice, but further mechanistic validation is required. Admittedly, the lack of a positive pharmacological control represents a limitation of this study. Future studies incorporating positive control groups will be performed to further validate the hypolipidemic effects of kombucha soymilk.

## 5 Conclusion

Kombucha soymilk consumption improved several hyperlipidemia-related phenotypes in high-fat diet mice and was associated with changes in specific gut microbial taxa and metabolic pathways. These findings support the potential of kombucha-fermented soymilk as a functional dietary intervention, while the mechanistic interpretation should remain cautious and requires further validation.

## Data Availability

The raw data supporting the conclusions of this article will be made available by the authors, without undue reservation.
