# Supplementary material for: Effect of kombucha soymilk on high fat diet mice: integrated insights from gut microbiome and metabolome analyses
Source: Front Nutr. 2026 May 13;13:1815305. doi: 10.3389/fnut.2026.1815305 (PMC13212210; doi:10.3389/fnut.2026.1815305)
Supplement: Supplementary file 1 [file Data_Sheet_1.PDF]

Supplementary data:

Table S1 Results of body weight in mice after modeling

	Initial body weigh t (g/mouse）	Final body weight (g/mouse）	Body weight gain	Serum-TC (mmol/L)	Serum-TG (mmol/L)
NC	22.00 ± 2.00	43.61± 1.20	22.61%	2.42 ± 0.0153	0.25 ± 0.0200
HM		53.47 ± 1.50		3.92 ± 0.1756$^{\#\#}$	0.76 ± 0.0208$^{\#\#}$

Note: Statistical analysis was performed using independent-sample t-test. ## $p < 0.01$, NC versus HM.

Table S2 Comparison of TC and TG Levels in Mice Among Different Groups

	B2	HM Baline	B5	B1	NC Basiline
TC	5.47 ± 0.2082^{A}	3.92 ± 0.1756^{B}	2.85 ± 0.1323^{C}	2.47 ± 0.0058^{D}	2.42 ± 0.0153^{D}
TG	0.95 ± 0.1002^{A}	0.76 ± 0.0208^{B}	0.65 ± 0.0503^{C}	0.22±0.0252^{D}	0.25 ± 0.0200^{D}

Note: Data were analyzed by one-way ANOVA followed by post hoc test. Different uppercase letters indicate highly significant differences ($p < 0.01$).
